# Glycosylation of Volatile Phenols in Grapes following Pre-Harvest (On-Vine) vs. Post-Harvest (Off-Vine) Exposure to Smoke

**DOI:** 10.3390/molecules26175277

**Published:** 2021-08-31

**Authors:** Julie A. Culbert, WenWen Jiang, Renata Ristic, Carolyn J. Puglisi, Elizabeth C. Nixon, Hongmei Shi, Kerry L. Wilkinson

**Affiliations:** 1 The Australian Wine Research Institute, P.O. Box 197, Glen Osmond, SA 5064, Australia; julie.culbert@awri.com.au (J.A.C.); maddy.jiang@awri.com.au (W.J.); 2 Waite Research Institute, School of Agriculture, Food and Wine, The University of Adelaide, PMB 1, Glen Osmond, SA 5064, Australia; renata.ristic@adelaide.edu.au (R.R.); carolyn.puglisi@adelaide.edu.au (C.J.P.); eclairenixon@gmail.com (E.C.N.); shhmchina@126.com (H.S.); 3 The Australian Research Council Training Centre for Innovative Wine Production, PMB 1, Glen Osmond, SA 5064, Australia; 4 Shandong Academy of Grape, Jinan 250100, China

**Keywords:** acid hydrolysis, guaiacol, smoke taint, syringol, wine

## Abstract

Taint in grapes and wine following vineyard exposure to bushfire smoke continues to challenge the financial viability of grape and wine producers worldwide. In response, researchers are studying the chemical, sensory and physiological consequences of grapevine smoke exposure. However, studies involving winemaking trials are often limited by the availability of suitable quantities of smoke-affected grapes, either from vineyards exposed to smoke or from field trials involving the application of smoke to grapevines. This study compared the accumulation of volatile phenol glycosides (as compositional markers of smoke taint) in Viognier and Cabernet Sauvignon grapes exposed to smoke pre- vs. post-harvest, and found post-harvest smoke exposure of fruit gave similar levels of volatile phenol glycosides to fruit exposed to smoke pre-harvest. Furthermore, wines made from smoke-affected fruit contained similar levels of smoke-derived volatile phenols and their glycosides, irrespective of whether smoke exposure occurred pre- vs. post-harvest. Post-harvest smoke exposure therefore provides a valid approach to generating smoke-affected grapes in the quantities needed for winemaking trials and/or trials that employ both chemical and sensory analysis of wine.

## 1. Introduction

When bushfires or prescribed burns occur near wine regions, vineyard exposure to smoke can affect the composition and sensory properties of grapes and wine [1], depending on the timing and duration of smoke exposure [2,3]. Wines made from smoke-exposed grapes that exhibit unpalatable ‘smoky’, ‘medicinal’ and ‘cold ash’ aromas and flavors, and/or an ‘ashy’ aftertaste, are considered to be ‘smoke tainted’ [4,5,6,7]. Volatile phenols, including guaiacol, 4-methylguaiacol, *o*-, *m*-, and *p*-cresol, syringol and 4-methylsyringol, are constituents of smoke [8,9,10], and have subsequently been detected in wines made from smoke-exposed grapes [2,3,4,5,6,7,10,11]. Previous studies have shown that smoke-derived volatile phenols accumulate in grapevine leaves and fruit in bound or precursor forms, i.e., they are rapidly metabolized, and produce volatile phenol glucosides, pentose-glucosides, glucose-glucoside (gentiobiosides), rutinosides and trisaccharides [12,13,14,15,16,17,18]. During winemaking, these glycosides can be extracted from grape skins and pulp, and hydrolyzed to release volatile phenols into the resulting wine [6,11,17,18,19]. However, a significant pool of volatile phenol glycosides is still present after fermentation [6,11,17,18,19] and are thought to contribute to the “ashy” aftertaste associated with smoke taint [20]. Glycosides are relatively stable at wine pH and ambient temperature [21,22,23], with only small changes in volatile phenol levels being observed in smoke tainted wines following 5–6 years of bottle aging [23].

Smoke-derived volatile phenols and their glycosides have become important chemical markers of grapevine smoke exposure. Several studies have evaluated direct and/or indirect methods for determining free and bound volatile phenol concentrations in grapes and/or wine [12,24,25,26,27,28]. Some of these methods are now offered by commercial laboratories, enabling grape and wine producers to screen fruit from vineyards that might have been exposed to smoke, i.e., to assess the viability of proceeding with harvest and winemaking. In contrast, these methods are used by researchers to study: the factors that influence the occurrence and intensity of smoke taint, e.g., grapevine phenology [2,3], grape variety [11,24], fruit maturity at harvest [29], and winemaking practices [6,30]; as well as strategies for prevention and/or amelioration of smoke taint, e.g., partial defoliation of grapevines [31], foliar applications of kaolin [16] or biofilm [32,33], washing grapes during or after smoke exposure [17,19], post-harvest ozonation [34,35], and the addition of fining agents to wine [36,37].

It is difficult for researchers to study the direct effects of grapevine exposure to bushfire smoke, given the occurrence of bushfires cannot be reliably predicted, and the inherent occupational health and safety risks associated with fires. While some studies have made use of fruit and/or wine samples arising from vineyards that have been exposed to bushfire smoke (e.g., [18,26,36,37,38,39]), smoke taint research often relies on grapevine exposure to smoke under experimental conditions, i.e., grapevines being enclosed in purpose-built smoke tents and exposed to smoke derived from the combustion of model fuels, in controlled and replicated experiments. Kennison and coworkers were the first to employ this approach, in field trials that demonstrated the timing and duration of smoke exposure influences the intensity of smoke taint in wine [2,3], and this also enabled the release of volatile phenols during fermentation of must from smoke-affected grapes to be studied [5]. It should be noted, however, that in early smoke taint literature (i.e., publications pre-2010), only volatile phenols were measured as markers of smoke taint, as the in vivo glycosylation of volatile phenols, and thus, existence of volatile phenol glycosides, had not yet been established.

One limitation associated with the use of ‘smoke tents’ is the number of grapevines that can be exposed to smoke in a single growing season, and therefore, the quantity of smoke-exposed fruit that can be generated for subsequent use in winemaking trials, or trials involving the amelioration of smoke taint in wine. This impacts both the number and scale of treatments that can be evaluated, as well as the availability of suitable volumes of wine for sensory analysis. As such, this study sought to compare the accumulation of volatile phenol glycosides in grapes exposed to smoke pre- vs. post-harvest, i.e., smoke exposure of bunches that remained on the vine vs. excised bunches, to establish whether post-harvest smoke exposure of fruit (which can more readily be performed at scale) generates fruit suitable for research purposes. Kennison and colleagues found post-harvest smoke exposure of grapes yielded wines containing elevated levels of smoke-derived volatile phenols [4], but until now, glycosylation of smoke-derived volatile phenols in excised fruit has not been studied. 

## 2. Results and Discussion

A range of analyses were applied to grapes, wine and/or acid hydrolysates of wine to determine the effects of bunch excision and smoke exposure. Berry weight was monitored following the application of experimental treatments and with the exception of excised Viognier bunches at t = 1, the mass of smoke-affected grapes was found to be significantly higher than for the corresponding control grapes, at each time-point (Table 1). This was unexpected, given previous studies found smoke exposure did not affect berry development or ripening [11,16], however, it is not clear if this can be directly attributed to smoke exposure. Whereas no significant differences were observed amongst the total soluble solids (TSS) levels of Viognier grapes at maturity (i.e., t = 7), grapes from excised Cabernet Sauvignon bunches had 5–6% lower TSS levels than grapes from bunches that remained on the vine (Table 1). This likely reflects sugar accumulation due to ripening in attached (on-vine) bunches. However, the observed changes in berry weight or TSS were not considered to be meaningful.

The volatile phenol levels of control and smoke-exposed grapes were also measured (Table S1). Low levels of guaiacol and cresols (i.e., ≤2.3 µg/kg) were detected in smoke-exposed Viognier grapes at t = 7 (maturity), with slightly higher levels (i.e., ≤3.7 µg/kg) observed at t = 1; while 1.3 µg/kg of *o*-cresol was detected in Cabernet Sauvignon grapes following pre-harvest smoke exposure, but only at t = 1. These results are consistent with findings from recent studies [16,17,19] and support the suggestion that metabolism of volatile phenols occurs rapidly after smoke exposure [16,17].

**Figure 1:**
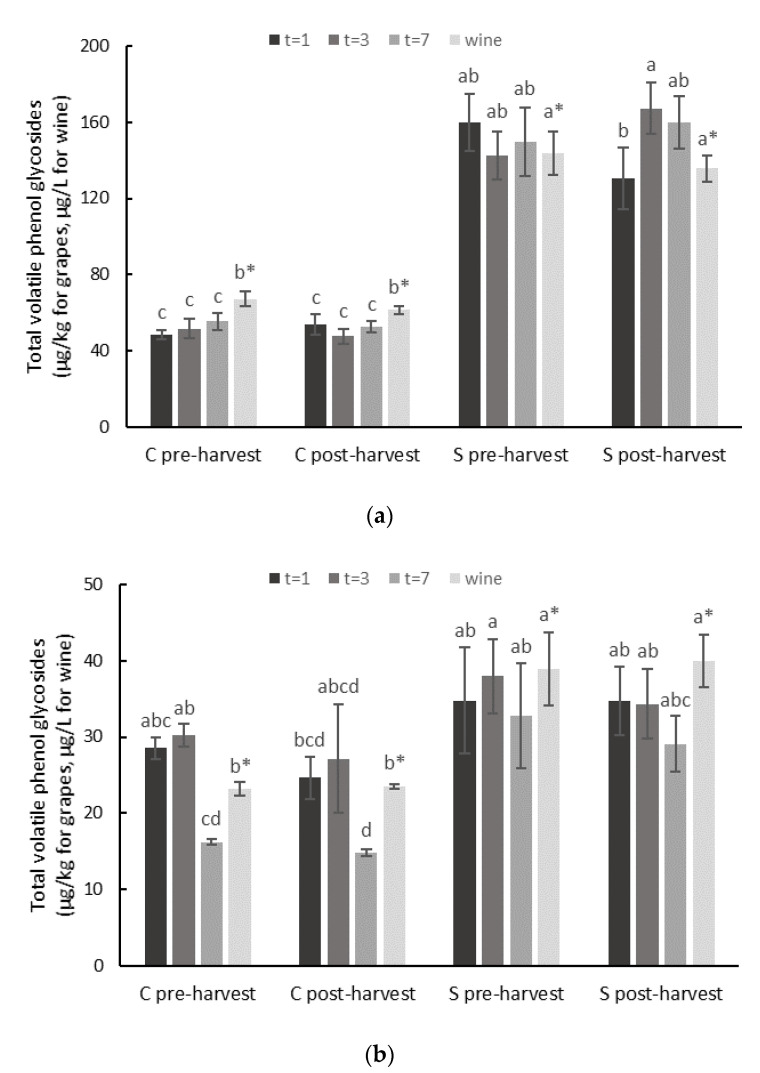
Total volatile phenol glycoside concentrations in control (C) and smoke-affected (S) (**a**) Viognier and (**b**) Cabernet Sauvignon grapes (in µg/kg, at 1, 3 and 7 days after smoke exposure) and wine (in µg/L). Values are means of three replicates (*n* = 3) measured as syringol glucose-glucoside equivalents. Different letters indicate statistical significance (*p* = 0.05, one-way ANOVA) amongst grape or wine (*) samples.

Background (naturally-occurring) levels of volatile phenol glycosides were detected in both Viognier and Cabernet Sauvignon grapes. Total glycoside levels ranged from 45 to 53 µg/kg for control Viognier grapes and from 11 to 27 µg/kg for control Cabernet Sauvignon grapes (Figure 1), with similar glycoside profiles observed irrespective of whether bunches remained on the vine or were excised (Table S2). Volatile phenol glycosides were also detected in control Viognier and Cabernet Sauvignon wines, at 59–64 and 20 µg/L, respectively (Figure 1, Table S3). Again, bunch excision did not affect glycoside levels.

Smoke exposure resulted in approximately two to three-fold higher levels of volatile phenol glycosides in Viognier grapes and wines (compared with corresponding controls), i.e., 128–165 µg/kg and 134–142 µg/L, respectively (Figure 1, Tables S2 and S3). In contrast, the glycoside levels detected in smoke-exposed Cabernet Sauvignon grapes were considerably lower (than smoke-exposed Viognier grapes), at 26–34 µg/kg (Figure 1, Table S2). This was attributed to Cabernet Sauvignon grapes being exposed to less dense smoke than Viognier grapes (due to windy conditions affecting retention of smoke in the tents during the Cabernet Sauvignon field trials). As such, the glycoside levels observed in smoke-exposed Cabernet Sauvignon grapes were only significantly different from their corresponding control grapes at t = 7 (i.e., at maturity). Nevertheless, the volatile phenol glycoside levels observed in Cabernet Sauvignon wines made from smoke-exposed grapes were significantly higher than the levels observed in control Cabernet Sauvignon wines (Figure 1, Table S3).

The most abundant volatile phenol glycosides detected in smoke-exposed Viognier grapes were pentose-glucosides of guaiacol, cresol and phenol, and the glucose-glucoside (gentiobioside) of syringol (Table S2), in agreement with previous studies [13,16,17,27]. Smoke exposure of Cabernet Sauvignon grapes was largely reflected by increased levels of glucose-glucosides of syringol and the presence of glucose-glucosides of 4-methylsyringol, albeit small but statistically significant increases were also observed for cresol rutinoside and phenol pentose-glucoside concentrations (Table S2). Differences in glycoside concentrations for smoke-exposed Viognier and Cabernet Sauvignon grapes can again be attributed to differences in the density of smoke applications achieved during field trials, but might also be due to grape variety. 

A recent study reported a delay in the accumulation of volatile phenol glycosides in Cabernet Sauvignon grapes following grapevine exposure to smoke [17]. Volatile phenols were observed at elevated concentrations in grapes sampled 1 h after smoke exposure, but 24 h after smoke exposure, levels had decreased by up to 75%. Although volatile phenol glycosides were detected in grapes sampled 24 h and/or 1 week after smoke exposure, substantial increases were observed in the grape glycoside levels detected at 1 vs. 4 weeks after smoke exposure. The authors suggested this might be explained by sequestration of volatile phenols, the presence of intermediates in the glycosylation pathway or by alternate volatile phenol storage forms. In the current study, only small temporal changes in grape glycoside levels were observed, and only in smoke-exposed Viognier grapes (Table S2). This suggests berry maturity at the time of smoke exposure might influence the accumulation and/or extractability of volatile phenol glycosides.

Significant differences were observed between the glycoside profiles of wines made from control and smoke-exposed grapes (Figure 1, Table S3). Background levels of pentose-glucosides of guaiacol, cresol and phenol were detected in control Viognier wine (i.e., 9–26 µg/L), but the concentrations of these glycoconjugates were significantly higher as a consequence of fruit exposure to smoke, along with several of the other volatile phenol glycosides that were measured, including syringol glucose-glucoside (Table S3). Lower background glycoside levels were observed in control Cabernet Sauvignon wines (i.e., ≤5.5 µg/L), with small, but statistically significant increases observed for several volatile phenol glycosides due to fruit exposure to smoke; again, this included syringol glucose-glucoside (Table S3). Differences in the density of smoke applications in part explain the differences observed in glycoside concentrations for smoke-affected Viognier and Cabernet Sauvignon wines, but variation in the background levels of glycosides for each variety was also a factor.

**Figure 2:**
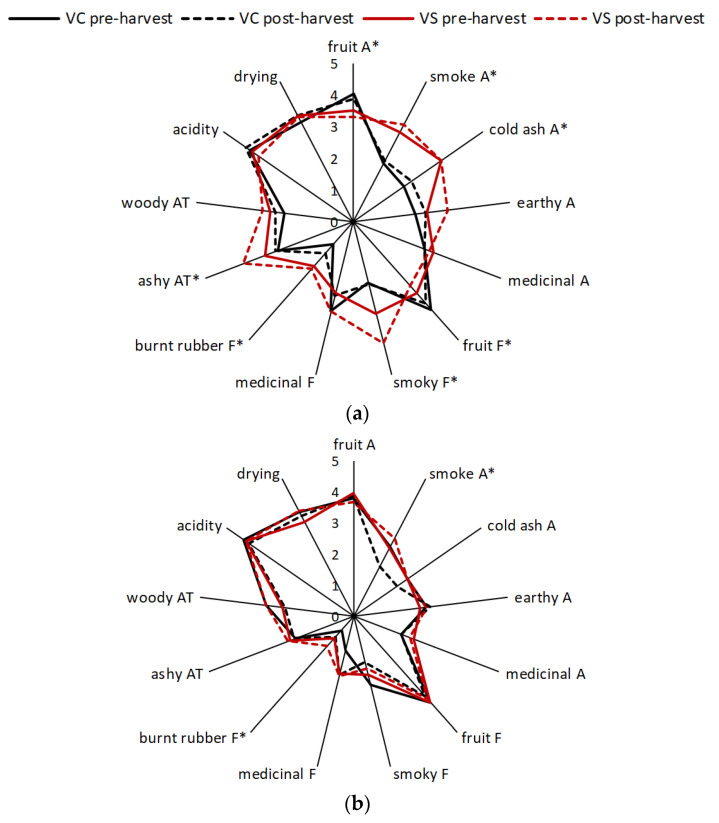
Sensory profiles of control (C) and smoke-affected (S) (**a**) Viognier and (**b**) Cabernet Sauvignon wines; A = aroma; F = flavor; AT = aftertaste. Values are mean intensity ratings of one wine per treatment, presented to 30 panelists; * denotes attributes for which ratings were statistically significant (*p* = 0.05, two-way ANOVA).

Guaiacol was the only volatile phenol detected (at ~1 µg/L) in control Viognier wines, while syringol was present (at 5 µg/L) in control Cabernet Sauvignon wines (Table 2); background volatile phenol levels were not affected by bunch excision. Viognier wines made with smoke-exposed grapes contained volatile phenols at concentrations 1–6 µg/L higher than their corresponding control wines, whereas the presence of guaiacol and *o*-cresol (at 1 µg/L each) in wines made from smoke-exposed Cabernet Sauvignon grapes provided compositional evidence of smoke exposure (Table 2). Sensory analysis of wines showed good agreement with chemical analysis results (Figure 2, Table S4). Significant differences were observed between the sensory profiles of control and smoke-affected Viognier wines (Figure 2a), with smoke-affected wines exhibiting diminished fruit aroma and flavor, and enhanced smoke and cold ash aromas, and smoky flavor, compared with their corresponding control wines. The smoke-affected Viognier wine made with grapes exposed to smoke post-harvest (i.e., excised bunches) was perceived as having a more intense smoky flavor and ashy aftertaste than the wine made with grapes that remained on the vine during smoke exposure (Table S4). Fewer sensory differences were perceivable amongst the Cabernet Sauvignon wines (Figure 2b), again reflecting the difference in smoke treatment of Viognier and Cabernet Sauvignon grapevines. The only statistically significant differences that were observed were for the intensity of smoke aroma, hotness, and burnt rubber flavor (Table S4), but these differences were not directly attributable to either smoke treatment or bunch excision. 

Evidence of smoke exposure was more apparent when the volatile phenol concentrations of wines were compared after acid hydrolysis (Table 2). Acid hydrolysis of control Viognier wines released 1–7 µg/L of guaiacol, 4-methylguaiacol, cresols and syringol, but no 4-methylsyringol. Significantly higher levels of these volatile phenols were observed when smoke-affected Viognier wines were subjected to acid hydrolysis (Table 2). Guaiacol and syringol levels increased the most, with approximately 2–3-fold and 6–8-fold increases being observed, respectively. A small (1.6 µg/L), but statistically significant difference was observed in the concentration of 4-methylsyringol detected after acid hydrolysis of smoke-affected Viognier wines, but this was the only compositional difference observed that could be attributed to excision of Viognier bunches prior to smoke exposure. There was no significant difference in the amounts of guaiacol or cresols detected after acid hydrolysis of Cabernet Sauvignon wines (Table 2). However, acid hydrolysis gave 1.6–1.8-fold higher syringol concentrations in smoke-affected Cabernet Sauvignon wines compared with their corresponding control wines, as well as small amounts (~2 µg/L) of 4-methylsyringol (Table 2). No compositional differences were apparent due to post-harvest (off vine) smoke exposure of Cabernet Sauvignon bunches. 

To further investigate the uptake and glycosylation of volatile phenols by excised bunches, a trial involving exposure of excised Viognier and Cabernet Sauvignon bunches to a subset of volatile phenols (in gaseous form) was undertaken. Grape bunches were placed in a 156 L glass chamber, together with an aqueous volatile phenol solution (comprising 250 mg/L each of guaiacol, *o*- and *m*-cresol, syringol and 4-methylsyringol), for 60 h. Low levels (up to 2.0 µg/kg) of guaiacol and *o*-cresol were detected in control grapes (Table 3), together with: 15 and 7.9 µg/kg of guaiacol and cresol pentose glucosides respectively, in control Viognier grapes; and 2.0–3.8 µg/kg of guaiacol and cresol pentose glucosides and syringol glucose glucoside, in control Cabernet Sauvignon grapes. However, guaiacol, *o*- and *m*-cresol, syringol and 4-methylsyringol were detected at varying levels (from 3.7–1079 µg/kg in free (aglycone) forms and from 17 to 2114 µg/kg in glycosylated forms) in treated grapes (Table 3), demonstrating both the uptake and glycosylation of volatile phenols by excised bunches, in agreement with findings from a similar, recent study [40]. 

Interestingly, guaiacol and *o*- and *m*-cresol were detected in treated grapes (in free and glycosylated forms) at considerably higher concentrations than syringol and 4-methylsyringol. This likely reflects relative differences in volatile phenol concentrations in the gas phase, as a consequence of their different physical properties, i.e., a combination of differences in molecular weight, water solubility, boiling point and vapor pressure. Significant variation was also observed in both volatile phenol and volatile phenol glycoside levels between the two grape varieties (Table 3) and between the bunch replicates for each variety (data not shown). This is likely explained by differences in bunch architecture, (e.g., bunch weight, berry number and size, bunch compactness, volume and/or surface area), as well as variation in changes in bunch weight during treatment (Table S5). Nevertheless, compositional analysis confirmed adsorption of gaseous volatile phenols by excised bunches, and their subsequent glycosylation. Furthermore, the concentrations of free and glycosylated volatile phenols achieved in grapes via post-harvest exposure to gaseous volatile phenols were substantially higher than the concentrations observed following post-harvest exposure to smoke. As such, this approach could be used to conduct preliminary or model experiments on small scale, prior to completion of more time and labor-intensive field trials.

The results from this study suggest the uptake and subsequent glycosylation of volatile phenols by grapes is not affected by excision of bunches prior to smoke exposure. As such, post-harvest smoke exposure could be employed to produce smoke-tainted grapes in the quantities needed for larger-scale, replicated winemaking trials and/or trials comprising multiple experimental treatments (e.g., fining trials), enabling both chemical and sensory analysis of wine; especially in years where commercial fruit or wine affected by bushfire smoke is not available. The density and/or duration of smoke exposure could be increased to obtain more heavily tainted grapes and wine, with higher levels of volatile phenols and their glycoconjugates, and more intense smoke-related sensory attributes. The only real limitation of this approach will be the need to apply smoke close to maturity, given grapes are non-climacteric and do not continue to ripen once removed from the grapevine. Model experiments involving exposure of excised bunches to one or more volatile phenols (in gaseous form) in lieu of smoke could also be undertaken. Again, the concentration of volatile phenols and duration of exposure can be manipulated to influence to what extent grapes are tainted.

In the current study, grape bunches were exposed to smoke immediately after harvest, while exposure to volatile phenols commenced within ~6 h of harvest, because grape metabolic processes, including glycosylation by glucosyltransferase enzymes, were expected to decline progressively following harvest. However, results from a recent study suggest glycosylation may continue for some time post-harvest. Bound volatile phenols were observed in table grapes 24 h after they were exposed to smoke [33], despite the table grapes being purchased from a grocer and presumably being subjected to some (unknown) period of delay between harvest and smoke exposure due to transportation and storage. The increase in volatile phenol concentrations observed following acid hydrolysis of the smoke-exposed table grapes indicates some metabolic processes persisted after harvest. While further research is needed to establish temporal changes in grape glucosyltransferase activity post-harvest, the cited study also proposes post-harvest exposure of grapes to smoke as an effective model for smoke taint research in the laboratory [33].

## 3. Materials and Methods

### 3.1. Chemicals

Chemicals (analytical and food grade) were purchased from Sigma Aldrich (Steinheim, Germany and Castle Hill, NSW, Australia) and solvents (HPLC grade) were purchased from Merck (Darmstadt, Germany). Deuterium-labelled internal standards (i.e., *d*_3_-guaiacol, *d*_3_-4-methylguaiacol, *d*_7_-*o*-cresol, *d*_3_-syringol and d_3_-syringol gentiobioside) were synthesized in house, as previously reported [13,27,41].

### 3.2. Field Trials

Field trials were conducted in a vineyard located at the University of Adelaide’s Waite Campus in Urrbrae, South Australia (34°58′ S, 138°38′ E) using Viognier and Cabernet Sauvignon grapevines (*Vitis vinifera*). Grapevines were: planted (in 1998) in north-south aligned rows on their own roots; trained to a bilateral cordon, vertical shoot positioned trellis system; hand-pruned to a two-node spur system; and drip irrigated. Three adjacent grapevines were exposed to straw-derived smoke (for 1 h), 7 days before maturity, using a purpose-built smoke tent (6.0 × 2.5 × 2.0 m) and experimental conditions described previously [2,3,11]. Immediately prior to smoke exposure, alternating fruit clusters were excised and re-suspended in the canopy fruit zone (i.e., from the fruiting wire, using bulldog clips), as close as possible to their original position. Fruit clusters were similarly excised and re-suspended in the canopy of three adjacent control vines (that were not exposed to smoke). Control vines were separated from smoke-exposed vines by a buffer panel of vines. The following day, excised bunches were collected from control and smoke-exposed vines and stored in darkness at 15 °C; with fruit from experimental treatments and replicate vines kept separate. 

Samples (100 berries per replicate per treatment, chosen randomly according to a previously published sampling protocol [42]) were collected at three time points: (i) 1 day after smoke application, (t = 1); (ii) 3 days after smoke application, (t = 3); and (iii) 7 days after smoke application (t = 7), i.e., harvest. The average berry weight of samples were determined before homogenization (T18 Ultra Turrax, IKA, Staufen, Germany); with TSS measured using a digital refractometer (PAL-1, Atago, Tokyo, Japan) at harvest (i.e., t = 7). Berry homogenates were then frozen at −4 °C until quantitation of volatile phenols and volatile phenol glycoconjugates (at approximately 1 month after sampling). The remaining control and smoke-exposed fruit was harvested 7 days after smoke exposure, when TSS levels were 23–24 °Brix, for winemaking.

### 3.3. Winemaking

Bunches were de-stemmed by hand and 1 kg of fruit (per replicate per treatment) crushed with the addition of 50 mg/L sulfur dioxide (added as a 10% solution of potassium metabisulphite (PMS)). Tartaric acid was added to adjust the pH of must to 3.0 and 3.5 for Viognier and Cabernet Sauvignon, respectively, prior to inoculation with 300 ppm of PDM yeast (Maurivin, Australia) and addition of diammonium phosphate (150 mg/L). White and red musts were fermented on skins (to maximize extraction of volatile phenol glycoconjugates) at ambient temperature (22–23 °C) for 7 days, with the cap plunged twice daily. Wines were then pressed and held at 20 °C until completion of fermentation (i.e., until residual sugars were <2 g/L), after which they racked from gross lees and cold stabilized (at 0 °C for 1 week). No wines underwent malolactic fermentation. Wine pH and free SO_2_ were adjusted to 3.5 and 30 mg/L respectively, before bottling (under screw cap closures). Bottles were stored at 15 °C for six months prior to chemical analysis.

### 3.4. Preparation of Acid Hydrolysates

Wines were subjected to strong acid hydrolysis according to methodology described previously [5,21]. Briefly, aliquots of control and smoke tainted wines (10 mL) were pH adjusted to 1.0 (via dropwise addition of concentrated sulfuric acid) and heated at 100 °C for 1 h. Hydrolysates were cooled to ambient temperature, pH adjusted back to wine pH (via dropwise addition of 1M aqueous sodium acetate) and frozen prior to chemical analysis. 

### 3.5. Laboratory Trials

An additional trial involving post-harvest exposure of grapes to volatile phenols (in gaseous form) was also performed. Three bunches of Viognier grapes and three bunches of Cabernet Sauvignon grapes (sourced from the same vineyard used for field trials, at maturity, i.e., at TSS levels of 23.7 and 21.3 °Brix, respectively) were placed in a 156 L glass chamber (91 × 38 × 45 cm), together with six glass dishes each containing 20 mL of an aqueous solution of volatile phenols (comprising ~250 mg/L each of guaiacol, *o*- and *m*-cresol, syringol and 4-methylsyringol). After 60 h of treatment (which commenced within 6 h of harvest), the grapes from each cluster were rinsed (with water), dried, weighed and homogenized (as above), prior to chemical analysis. Bunch weights were obtained before and after treatment; berry numbers were also counted prior to homogenization. Control grapes (3 bunches per variety) were similarly harvested, weighed and homogenized for chemical analysis. The volume of aqueous volatile phenol solution remaining after treatment was measured and ranged from 3.6 to 7.4 mL per dish (and 32.2 mL in total).

### 3.6. Chemical Analysis

The concentrations of volatile phenols (guaiacol, 4-methylguaiacol, *o*-, *m*- and *p*-cresol, syringol and 4-methylsyringol) were measured in grapes, wine and acid hydrolysates of wine, by the Australian Wine Research Institute’s (AWRI) Commercial Services Laboratory (Adelaide, SA, Australia). Volatile phenols were measured using an Agilent 6890 gas chromatograph coupled to a 5973 mass selective detector (Agilent Technologies, Forest Hill, Vic., Australia), using stable isotope dilution analysis (SIDA) methods described previously [13,41]; with the method developed for analysis of wine used for acid hydrolysates. The concentrations of volatile phenol glycosides were also measured in grapes, wine and acid hydrolysates of wine, as syringol glucose-glucoside (gentiobioside) equivalents, using liquid chromatography-tandem mass spectrometry (LC-MS/MS) according to previously published SIDA methods [13]; again, the method developed for analysis of wine was used for analysis of acid hydrolysates. Glycoconjugate analyses were performed on an Agilent 1200 high performance liquid chromatograph (HPLC) equipped with a 1290 binary pump coupled to an AB SCIEX Triple Quad^TM^ 4500 tandem mass spectrometer, with a Turbo V^TM^ ion source (Framingham, MA, USA). Data acquisition and processing were performed using Analyst software (version 1.7 AB SCIEX). The preparation of isotopically labelled internal standards, method validation and instrumental operating conditions were as previously reported [13,27]. The limit of quantitation for volatile phenols and volatile phenol glycosides was 1–2 and 1 µg/L, respectively. 

### 3.7. Sensory Analysis

The replicate wines from each treatment were evaluated (by two sensory experts with >10 years of experience working with smoke tainted wines) to ensure there were no obvious faults or sensory differences amongst the replicates. The wine replicates from each treatment were then blended and their sensory profiles determined (using the rate-all-that-apply (RATA) method [43]) with a panel comprising staff and students from the University of Adelaide and AWRI, and regular wine consumers (*n* = 30, 8 male and 22 female, aged between 25 and 67 years). Prior to wine evaluation, panelists completed a brief induction, during which they were introduced to both the RATA procedure and a list of attributes adapted from previous studies [11,23]. RATA assessments were conducted in sensory booths at 22–23 °C under sodium lights, with wine aliquots (25 mL) presented monadically, in a randomized order, in covered, 3-digit coded 215 mL stemmed wine glasses. Panelists rated the intensity of each sensory attribute using line scales (where 0 = “not perceived”, 1 = “extremely low”, 4 = “moderate” and 7 = “extremely high”). Panelists rinsed with water between samples and were provided plain crackers as palate cleansers. To minimize sensory fatigue and potential carryover of sensory effects between samples, 1 min breaks were enforced between samples, with a 5 min break enforced between the brackets of white and red wine. Data were acquired with Red Jade software (Redwood Shores, CA, USA).

### 3.8. Data Analysis

Chemical data were analyzed by analysis of variance (ANOVA) using GenStat (19th Edition, VSN International Limited, Herts, UK). Mean comparisons were performed by least significant difference (LSD) multiple comparison test at *p* < 0.05. Sensory data were analyzed by two-way ANOVA using participants as a random factor and wines as a fixed factor, with Fischer’s LSD post-hoc test (*p* ≤ 0.05) to determine significant differences between the treatments at *p* < 0.05, using XLSTAT (version 2018.1.1., Addinsoft, New York, NY, USA).

## Figures and Tables

**Table 1:** Berry weight and total soluble solids (TSS) of control (C) and smoke-exposed (S) grapes.

Treatment			Berry Weight (g)			TSS (°Brix)
			t = 1	t = 3	t = 7	t = 7
Viognier	C	pre-harvest	1.03 b	1.03 c	0.90 c	23.8
		post-harvest	1.07 b	1.09 c	0.99 bc	23.8
	S	pre-harvest	1.25 a	1.23 b	1.07 ab	23.5
		post-harvest	1.13 ab	1.36 a	1.19 a	22.9
	*p*		0.012	0.001	0.004	ns
Cabernet Sauvignon	C	pre-harvest	1.00 b	0.99 b	1.01 b	23.6 a
		post-harvest	0.99 b	0.96 c	0.92 c	22.2 bc
	S	pre-harvest	1.09 a	1.14 a	1.08 a	22.6 b
		post-harvest	1.10 a	1.16 a	1.03 ab	21.5 c
	*p*		0.009	<0.001	0.003	0.007

Values are means of three replicates (*n* = 3). Different letters (within columns, by variety) indicate statistical significance (*p* = 0.05, one way ANOVA) at different time points, i.e., 1 day after smoke exposure (t = 1); 3 days after smoke exposure (t = 3); and 7 days after smoke exposure (t = 7); ns = not significant.

**Table 2:** Concentrations of volatile phenols (µg/L) in wines (and acid hydrolysates of wine) made from control (C) and smoke-exposed (S) grapes.

Treatment			Wine					Acid Hydrolysates				
			Guaiacol	4-Methyl Guaiacol	Total Cresols	Syringol	4-Methyl Syringol	Guaiacol	4-Methyl Guaiacol	Total Cresols	Syringol	4-Methyl Syringol
Viognier	C	pre-harvest	1.3 b	nd	nd	nd	nd	7.3 b	1.3 b	4.3 b	4.7 b	nd
		post-harvest	1.0 b	nd	nd	nd	nd	7.7 b	1.3 b	4.3 b	6.3 b	nd
	S	pre-harvest	5.7 a	1.3	2.3	2.0	nd	20.7 a	4.4 a	9.7 a	37.0 a	6.7 b
		post-harvest	7.3 a	1.7	4.0	3.0	nd	18.3 a	4.7 a	12.3 a	38.3 a	8.3 a
	*p*		<0.001	ns	ns	ns	–	0.003	<0.001	0.002	<0.001	<0.001
CabernetSauvignon	C	pre-harvest	nd	nd	nd	5.0	nd	4.3	tr	4.7	14.7 b	nd
		post-harvest	nd	nd	nd	5.0	nd	4.0	tr	2.3	13.7 b	nd
	S	pre-harvest	1.0	nd	1.3	5.0	nd	4.3	tr	4.3	23.7 a	2.0
		post-harvest	1.0	nd	1.0	5.5	nd	5.5	tr	4.5	25.3 a	1.7
	*p*		ns	–	ns	ns	–	ns	–	ns	0.019	ns

Values are means of three replicates (*n* = 3); nd = not detected. Different letters (within columns, by variety) indicate statistical significance (*p* = 0.05, one way ANOVA); ns = not significant.

**Table 3:** Concentration of volatile phenols and their glycosides (µg/kg) in grapes exposed to volatile phenols for 60 h.

Treatment	Guaiacol	*o*-Cresol	*m*-Cresol	Syringol	4-Methy Syringol	GuPG	GuR	CrPG	CrR	SyrGG	4MSGG
control Viognier	2.0	1.0	nd	nd	nd	15	nd	7.9	nd	nd	nd
treated Viognier	227	605	472	9.7	3.7	1111	25	2114	205	45	17
control Cabernet Sauvignon	nd	1.3	nd	nd	nd	3.5	nd	3.8	nd	2.0	nd
treated Cabernet Sauvignon	426	1079	646	35	10	482	90	1196	571	102	26

Glycosides measured as syringol glucose-glucoside (gentiobioside) equivalents; nd = not detected. GuPG = guaiacol pentose-glucoside; GuR = guaiacol rutinoside; CrPG = cresol pentose-glucoside; CrR = cresol rutinoside; SyrGG = syringol glucose-glucoside; 4MSGG = 4-methylsyringol glucose-glucoside.

## Data Availability

The data presented in this study are available in Supplementary Materials.

## Supplementary Materials

The following are available online, Table S1: Concentrations of volatile phenols (µg/kg) in control (C) and smoke-exposed (S) grapes, sampled 7 days after smoke exposure. Table S2: Concentrations of volatile phenol glycosides (µg/kg) in control (C) and smoke-exposed (S) grapes, sampled 1, 3 or 7 days after smoke exposure. Table S3: Concentrations of volatile phenol glycosides (µg/L) in wines made from control (C) and smoke-exposed (S) grapes. Table S4: Mean intensity ratings for sensory attributes of Viognier and Cabernet Sauvignon wines made from control (C) and smoke-affected (S) grapes. Table S5: Bunch weights and berry numbers for Viognier and Cabernet Sauvignon grapes exposed to gaseous volatile phenols (for 60 h) post-harvest.

## Sample Availability

Samples of compounds are not available from the authors.
