# Supplementary material for: Glycosylation of Volatile Phenols in Grapes following Pre-Harvest (On-Vine) vs. Post-Harvest (Off-Vine) Exposure to Smoke
Source: Molecules. 2021 Aug 31;26(17):5277. doi: 10.3390/molecules26175277 (PMC8433723; doi:10.3390/molecules26175277)
Supplement: Supplementary file 1 [file molecules-26-05277-s001.zip › molecules-1310816-supplementary.pdf]

Supplementary Materials

Glycosylation of Volatile Phenols in Grapes Following Pre-Harvest (On-Vine) vs. Post-Harvest (Off-Vine) Exposure to Smoke

Julie A. Culbert [1], WenWen Jiang [1], Renata Ristic [2,3], Carolyn J. Puglisi [2], Elizabeth C. Nixon [2], Hongmei Shi [2,4] and Kerry L. Wilkinson [2,3,*]

[1] The Australian Wine Research Institute, PO Box 197, Glen Osmond, SA, 5064, Australia; julie.culbert@awri.com.au (J.C.); maddy.jiang@awri.com.au (W.J.);
[2] The University of Adelaide, School of Agriculture, Food and Wine, PMB 1, Glen Osmond, SA, 5064, Australia; renata.ristic@adelaide.edu.au (R.R.); carolyn.puglisi@adelaide.edu.au (C.P.); eclairenixon@gmail.com (E.N.)
[3] The Australian Research Council Training Centre for Innovative Wine Production, PMB 1, Glen Osmond, SA, 5064, Australia
[4] Shandong Academy of Grape, Jinan 250100, China, shhmchina@126.com (H.S.)
* Correspondence: kerry.wilkinson@adelaide.edu.au (K.W.); Tel.: +61-8-8313-7360

Table S1. Concentrations of volatile phenols (μg/kg) in control (C) and smoke-exposed (S) grapes, sampled 7 days after smoke exposure.

Treatment			Guaiacol	4-Methyl Guaiacol	Total Cresols	Syringol
Viognier	C	pre-harvest	(1.0) nd	nd	nd	nd
		post-harvest	nd	nd	nd	nd
	S	pre-harvest	(3.7) 2.3	nd	(1.3) 1.0	nd
		post-harvest	2.3	nd	1.3	nd
Cabernet Sauvignon	C	pre-harvest	nd	nd	nd	nd
		post-harvest	nd	nd	nd	nd
	S	pre-harvest	nd	nd	(1.3) nd	nd
		post-harvest	nd	nd	nd	nd

Values are means of three replicates (*n* = 3); nd = not detected. Values in parentheses are volatile phenols detected 1 day after smoke exposure (i.e., at t=1).

Table S2. Concentrations of volatile phenol glycosides (μg/kg) in control (C) and smoke-exposed (S) grapes, sampled 1, 3 or 7 days after smoke exposure.

	Treatment		Sample Time	GuG	GuGG	GuPG	GuR	4MGR	CrG	CrGG	CrPG	CrR	PhG	PhGG	PhPG	PhR	SyrG	SyrGG	SyrPG	4MSGG	Total
Viognier	C	pre-harvest	t = 1	1.2 d	tr	11.9	tr	tr	1.7 c	tr	9.4 d	1.8	tr	nd	6.9 c	tr	tr	1.4	11.1	tr	45
			t = 3	1.3 d	tr	12.9	tr	tr	1.9 c	tr	9.7 d	1.8	tr	nd	7.5 c	tr	tr	1.6	11.9	tr	48
			t = 7	tr	tr	23.5	tr	1.3	tr	tr	11.6 d	2.0	tr	nd	9.4 c	tr	tr	nd	4.1	1.0	53
		post-harvest	t = 1	1.1 d	tr	14.2	tr	tr	1.6 c	tr	10.0 d	1.8	tr	nd	7.5 c	tr	tr	1.5	12.7	tr	50
			t = 3	1.1 d	tr	12.3	tr	tr	1.7 c	tr	8.6 d	1.5	tr	nd	6.8 c	tr	tr	1.4	11.2	tr	45
			t = 7	tr	tr	22.1	tr	tr	tr	tr	11.2 d	1.8	nd	nd	9.0 c	tr	tr	nd	4.1	tr	48
	S	pre-harvest	t = 1	5.1 a	3.2	36.8	1.0	1.8	5.4 a	tr	28.3 a	2.8	tr	tr	22.2 ab	tr	tr	28.4	16.2	7.0	158
			t = 3	4.0 b	3.1	29.1	tr	1.2	3.4 b	tr	23.5 bc	2.6	tr	tr	20.2 b	tr	tr	34.7	11.5	7.0	140
			t = 7	1.5 d	4.0	39.7	tr	3.6	1.0 c	tr	20.9 c	3.0	tr	tr	19.1 b	tr	1.4	39.2	6.7	7.8	148
		post-harvest	t = 1	4.9 ab	3.0	27.7	tr	1.4	4.6 a	nd	21.4 c	2.1	tr	tr	18.5 b	tr	tr	27.0	11.3	6.3	128
			t = 3	5.3 a	4.0	35.0	tr	1.4	4.7 a	tr	26.7 ab	2.8	2.0	tr	25.0 a	tr	tr	37.9	11.4	9.1	165
			t = 7	2.7 c	4.5	42.8	tr	2.8	1.8 c	tr	23.1 ab	3.1	tr	tr	20.2 b	tr	2.1	39.9	6.1	8.8	158
	P	treatment		<0.001	ns	<0.001	–	<0.001	<0.001	–	<0.001	<0.001	–	–	<0.001	–	–	<0.001	ns	<0.001	–
		treatment x time		0.023	ns	ns	–	ns	0.008	–	0.017	ns	–	–	0.026	–	–	ns	ns	ns	–
Cabernet Sauvignon	C	pre-harvest	t = 1	tr	tr	2.3	2.5	tr	4.4	tr	4.1	1.4	tr	nd	3.8	tr	tr	3.0	4.2	tr	26
			t = 3	tr	tr	2.3	2.8	tr	4.6	tr	3.5	1.7	tr	tr	3.8	tr	tr	3.2	4.9	tr	27
			t = 7	tr	tr	2.8	tr	tr	tr	tr	2.5	1.6	tr	tr	2.7	tr	tr	2.1	tr	tr	12
		post-harvest	t = 1	tr	tr	2.0	2.2	tr	3.3	tr	3.4	1.3	tr	nd	3.0	tr	tr	3.0	4.0	tr	22
			t = 3	tr	tr	2.1	2.5	tr	4.5	tr	3.5	1.5	tr	nd	3.1	tr	tr	2.5	4.3	tr	24
			t = 7	tr	tr	2.6	tr	tr	tr	tr	2.4	1.5	tr	nd	2.6	tr	tr	1.9	tr	tr	11
	S	pre-harvest	t = 1	tr	tr	2.2	1.8	tr	3.4	tr	3.8	1.9	tr	tr	3.9	tr	tr	9.3	4.1	1.2	32
			t = 3	tr	tr	2.0	1.9	tr	3.1	tr	4.4	2.7	tr	tr	4.4	tr	tr	10.6	3.9	1.4	34
			t = 7	tr	tr	3.0	tr	1.0	tr	tr	2.8	3.1	tr	tr	3.3	1.3	tr	12.3	1.6	1.8	30
		post-harvest	t = 1	tr	tr	2.2	2.0	tr	5.0	tr	3.8	1.9	tr	tr	3.9	tr	tr	7.6	3.7	1.2	31
			t = 3	tr	tr	2.1	1.9	tr	3.3	tr	3.8	2.0	tr	tr	3.8	tr	tr	9.1	3.9	1.2	31
			t = 7	tr	tr	2.8	tr	1.0	tr	tr	2.9	2.8	tr	tr	3.7	1.1	tr	9.2	1.4	1.5	26
	P	treatment		–	–	ns	0.041	ns	ns	–	ns	0.003	–	–	0.033	ns	–	<0.001	ns	ns	–
		treatment x time		–	–	ns	ns	ns	ns	–	ns	ns	–	–	ns	–	–	ns	ns	ns	–

Sample times are days after smoke exposure. Values are means of three replicates (*n* = 3) measured as syringol glucose-glucoside equivalents; nd = not detected; tr = trace (i.e., 0.5–1 μg/kg). Different letters within a column (by variety) indicate statistical significance (P = 0.05, two-way ANOVA); ns = not significant. Gu = guaiacol; 4MG = 4-methylguaiacol; Cr = cresol; Ph = phenol; Syr = syringol; 4MS = 4-methylsyringol; G = glucoside; GG = glucose-glucoside; PG = pentose-glucoside; R = rutinoside.

Table S3. Concentrations of volatile phenol glycosides (μg/L) in wines made from control (C) and smoke-exposed (S) grapes.

		Treatment	GuG	GuGG	GuPG	GuR	4MGR	CrG	CrGG	CrPG	CrR	PhG	PhGG	PhPG	PhR	SyrG	SyrGG	SyrPG	4MSGG	Total
Viognier	C	pre-harvest	1.3 b	tr	26.2 b	tr	1.5 b	4.7	tr	12.9 b	2.7 b	1.0 b	tr	9.6 b	tr	tr	nd	4.1 b	tr	64
		post-harvest	1.1 b	tr	23.2 b	tr	1.2 b	4.6	tr	12.3 b	2.4 b	1.1 b	tr	9.1 b	tr	tr	nd	3.7 b	tr	59
	S	pre-harvest	3.4 a	tr	39.2 a	tr	4.4 a	5.5	tr	21.0 a	3.9 a	5.5 a	tr	18.0 a	1.2	3.0	27.0	6.6 a	3.2	142
		post-harvest	4.1 a	tr	38.2 a	tr	3.5 a	6.4	tr	20.3 a	3.8 a	6.1 a	tr	15.7 a	1.2	3.7	22.2	6.0 a	2.8	134
		P	<0.001	–	0.002	–	0.046	ns	–	0.004	0.004	<0.001	–	0.001	ns	ns	ns	0.002	ns	–
Cabernet Sauvignon	C	pre-harvest	tr	tr	2.4	2.3	tr	4.5 b	tr	2.4	1.6 b	1.2 b	tr	4.2 b	1.1 b	tr	nd	tr	tr	20
		post-harvest	tr	tr	2.3	2.0	tr	5.5 a	tr	2.4	1.4 b	1.1 b	tr	4.5 b	1.0 b	tr	nd	tr	tr	20
	S	pre-harvest	tr	tr	2.6	2.2	1.0	4.5 b	tr	2.7	2.8 a	3.2 a	tr	5.3 a	2.2 a	1.2	7.2	2.0	tr	37
		post-harvest	tr	tr	2.7	2.1	tr	5.8 a	tr	3.0	2.5 a	3.4 a	tr	5.8 a	2.0 a	1.1	6.5	1.5	tr	36
		P	–	–	ns	ns	–	0.001	–	ns	0.002	<0.001	–	0.002	<0.001	ns	ns	ns	–	–

Values are means of three replicates (n = 3) measured as syringol glucose-glucoside equivalents; nd = not detected; tr = trace (i.e. < 0.5–1 μg/kg). Different letters within a column (by variety) indicate statistical significance (p = 0.05, one-way ANOVA); ns = not significant. Gu = guaiacol; 4MG = 4-methylguaiacol; Cr = cresol; Ph = phenol; Syr = syringol; 4MS = 4-methylsyringol; G = glucoside; GG = glucose-glucoside; PG = pentose-glucoside; R = rutinoside.

Table S4. Mean intensity ratings for sensory attributes of Viognier and Cabernet Sauvignon wines made from control (C) and smoke-affected (S) grapes.

Attribute	Viognier					Cabernet Sauvignon				
	C		S		P	C		S		P
	Pre-harvest	Post-harvest	Pre-harvest	Post-harvest		Pre-harvest	Post-harvest	Pre-harvest	Post-harvest	
fruit aroma	4.0 a	3.9 ab	3.5 bc	3.3 c	0.010	3.8	3.9	4.0	3.7	ns
smoke aroma	2.1 b	2.2 b	3.2 a	3.5 a	<0.0005	2.5 a	1.8 b	2.5 ab	2.8 a	0.047
cold ash aroma	1.9 b	2.3 b	3.4 a	3.4 a	<0.0005	2.1	1.7	2.1	2.1	ns
earthy aroma	2.0	2.3	2.4	3.0	ns	2.4	2.5	2.1	2.4	ns
medicinal aroma	2.4	2.4	2.7	2.6	ns	1.6	1.6	2.1	1.9	ns
burnt rubber aroma	1.2	1.5	1.7	2.0	ns	0.7	1.0	1.2	1.1	ns
fruit flavor	3.7 a	3.5 a	3.0 b	2.7 b	<0.0001	3.7	3.5	3.7	3.6	ns
smoky flavor	2.0 c	2.0 c	3.0 b	4.0 a	<0.0001	2.3	1.5	1.9	1.7	ns
medicinal flavor	2.9	2.4	2.3	2.9	*ns*	1.2	1.9	1.9	2.0	ns
burnt rubber flavor	0.9 b	1.3 ab	1.9 a	2.0 a	0.029	0.6 b	0.9 ab	0.9 ab	1.3 a	0.033
ashy aftertaste	2.5 b	2.6 b	3.0 b	3.7 a	0.005	2.0	2.1	2.2	2.3	ns
woody aftertaste	2.2	2.5	2.6	2.9	ns	2.8	2.2	2.3	2.8	ns
metallic	1.8	1.7	1.3	1.4	ns	1.0	1.2	1.1	1.0	ns
acidity	4.0	4.1	3.9	3.6	ns	4.3	4.1	4.2	4.2	ns
hotness	3.8	3.4	3.6	3.8	ns	3.5 a	3.1 b	3.3 ab	3.1 b	0.047
bitterness	3.8	3.8	3.7	3.8	ns	3.3	3.3	3.2	3.5	ns
drying	3.5	3.8	3.8	3.7	ns	3.8	3.7	3.4	3.8	ns

Values are means for one wine per treatment presented to 30 judges. Different letters within rows (by variety) indicate statistical significance (P = 0.05, two-way ANOVA); ns = not significant.

Table S5. Bunch weights and berry numbers for Viognier and Cabernet Sauvignon grapes exposed to gaseous volatile phenols (for 60 hours) post-harvest.

Treatment		Bunch Weight (g)		Berry Number
		Pre-Treatment	Post-Treatment[a]	
control Viognier	bunch 1	126.94	–	98
	bunch 2	225.56	–	182
	bunch 3	186.73	–	150
treated Viognier	bunch 1	331.59	305.51 (7.9%)	247
	bunch 2	159.88	146.97 (8.1%)	126
	bunch 3	99.45	90.23 (9.3%)	71
control Cabernet Sauvignon	bunch 1	77.85	–	72
	bunch 2	120.97	–	142
	bunch 3	62.40	–	70
treated Cabernet Sauvignon	bunch 1	70.34	62.15 (11.6%)	87
	bunch 2	87.49	75.16 (14.1%)	110
	bunch 3	92.47	83.25 (10.0%)	103

[a]Percentage change in bunch weight shown in brackets.
